# Negotiating the Beginning of Care: A Grounded Theory Study of Health Services for Amyotrophic Lateral Sclerosis

**DOI:** 10.3390/brainsci12121623

**Published:** 2022-11-26

**Authors:** Sara Alquati, Luca Ghirotto, Ludovica De Panfilis, Cristina Autelitano, Elisabetta Bertocchi, Giovanna Artioli, Francesca Sireci, Silvia Tanzi, Simona Sacchi

**Affiliations:** 1 Palliative Care Unit, Azienda USL—IRCCS di Reggio Emilia, 42123 Reggio Emilia, Italy; 2 Qualitative Research Unit, Azienda USL—IRCCS di Reggio Emilia, 42123 Reggio Emilia, Italy; 3 Bioethics Unit, Azienda USL—IRCCS di Reggio Emilia, 42123 Reggio Emilia, Italy; 4 Department of Medicine and Surgery, University of Parma, 43125 Parma, Italy; 5 Neurology Unit, Azienda USL—IRCCS di Reggio Emilia, 42123 Reggio Emilia, Italy

**Keywords:** grounded theory, amyotrophic lateral sclerosis, health services, patient care planning

## Abstract

A range of professional figures are needed to preserve the quality of life of people with amyotrophic lateral sclerosis. This study aimed to explore the beginning of the care process as negotiated by people with amyotrophic lateral sclerosis, their caregivers, and healthcare professionals. We designed the study according to the constructivist Grounded Theory method, collecting data through open-ended, semi-structured interviews, employing theoretical sampling and constant comparison, and performing conceptual coding as data analysis. By naming the core category “off-beat interfacing”, we were able to show how the demands of the professionals concerned did not correspond to the ability of people with ALS and their proxies to process information, deal with requests, and be at ease in making decisions at the beginning of the shared care pathway. Three categories were generated: (i) navigating different paths, (ii) offering and experiencing a standard, non-personalized pathway, and (iii) anticipating decisions. The network of services must be organized according to guidelines, but must also contemplate a patient-family-centered approach that permits more personalized assistance.

## 1. Introduction

Amyotrophic lateral sclerosis (ALS) is a neurodegenerative disease predominantly affecting the motor neurons in the brain and spinal cord, leading to weakness of voluntary muscles [1]. Muscle weakness progresses gradually, usually causing respiratory failure [2]. Survival varies from several months to more than ten years [2]. About half of all people with ALS also suffer from cognitive and behavioral changes. The incidence of ALS is 1.9/100,000/year worldwide, and it is estimated that the number of individuals who have ALS will increase by 69% by 2040 [3,4]. Since no successful cures or preventive treatments are available in current clinical practice, therapy is primarily palliative. This approach focuses on alleviating symptoms and, depending on the course of the disease and the preferences of individuals with ALS, may include artificial ventilation and/or a feeding tube [5,6] to improve survival and health-related quality of life (QoL) [7,8].

ALS significantly impacts function, causing activity limitations and participation restrictions in many settings. Various healthcare services are therefore needed to preserve patient QoL [9]. Medical care and other healthcare and social services are necessary to support individuals with ALS and their families due to loss of autonomy (for example, special transport services, rehabilitation, housing, home care services, etc.). The need for services and special aids has increased over time [7].

How health and social services are organized differs internationally. However, the involvement and coordination of a multidisciplinary care team [5,8,10,11,12] is a standard pillar [7,13], and is also suggested by current guidelines [14]. This system of interdisciplinary care teamwork aims to improve the QoL and survival of individuals with ALS, and minimize the burden experienced by those individuals and their caregivers [15]. Literature has shown that caring for a person with ALS can produce high levels of distress, anxiety, and depression in caregivers (CGs) and, consequently, an impairment of the QoL of people within the ALS-CG system [16,17,18].

The diagnostic timelines have remained consistent over 20 years (1989–2008) [19], with delays in referral within primary and secondary care services [20,21]. Because diagnostic delay adds to the psychological stress of individuals with ALS and their caregivers (CGs) by causing additional anxiety and uncertainty [20], the starting point for health services may be crucial.

Indeed, initiating care is critical: healthcare professionals (HPs) should pay attention to the timing and communication of the diagnosis, the services to be accessed, and decisions concerning symptom management and end-of-life care [22].

When receiving the diagnosis and initiating the relationship with HPs, individuals with ALS and their CGs have broad expectations of healthcare services [23]. Complaints about inadequate emotional support [24], lack of continuity and coordination of services [25], unmet needs [26], and difficulties in accessing services [27] have been reported. In terms of service needs, in the Australian context, for example, patients testified to gaps in rehabilitation, while CGs criticized shortcomings in psychological support [28].

In this regard, studying satisfaction in service provisions could be seen as reductive when viewed in relation to the complex range of interactions that define that satisfaction [29]. The needs and expectations of individuals with ALS-CGs [28,30] are in dialogue with the perspectives and behaviors of HPs. Moreover, there can be no doubt that services must address multiple dynamics among individuals with ALS/CGs and HPs, which impact satisfaction, QoL, and well-being.

Values important to individuals with ALS and their CGs are not always aligned with those of healthcare providers [31], and a deeper understanding of the factors that are important to the service user could potentially improve service delivery engagement and efficiency [29].

In particular, the point where the use of the services begins, represented by the communication of the diagnosis, impacts the relationship between the actors within the care process. This phase is crucial, given that individuals with ALS often experience delays in diagnosis and initiating the care they need. Understanding the process underlying the early stages of care and the use of services may provide important information about improving relationships, trust, support, and the climate of respect. This study aimed to explore the beginning of the care process negotiated by individuals with ALS, their CGs, and HPs.

## 2. Materials and Methods

### 2.1. Methodological Approach

The generative research question was: “How is care for individuals with ALS initiated?”. This entails a psychosocial process in which the investigation is consistent with the Grounded Theory Method (GTM). We opted for Charmaz’s constructivist grounded theory method [32]: the constructivist stance of the method refers to how scientific knowledge is understood (an intersubjective construct influenced by participants, researchers, and the way they collect data [32]). By following this specific constructivist approach, we intended to explore the meaning attributed to phenomena (signified) and the (contextual and social) aspects negotiated by the informants. Constructivist GTM made it possible to develop an explicative model than can clarify which factors influence the use of social and health services. We reported the study by applying the Consolidated criteria for Reporting Qualitative Research [33].

### 2.2. Research Settings

The research was conducted at the General Hospital of Reggio Emilia, administered by the Local Health Authority for Reggio Emilia Province (which has a catchment area covering more than 530,000 inhabitants). Researchers involved the respiratory and neurology departments and the Hospital’s palliative care unit in participant recruitment.

### 2.3. Sampling and Recruitment

We performed the initial and theoretical sampling. The initial sampling was carried out using a purposive approach (and was, thus, driven by the purpose/aim of the study, hence individuals with ALS, CGs, and HPs). Individuals with ALS and CGs were considered eligible for recruitment in the study if they were 18 years or older, could provide written consent and participate in data collection, and had been diagnosed no earlier than three months before the interview. HPs must be involved in the care process regardless of their disciplines/professional backgrounds.

The principal investigator (S.A.) contacted the potential participants by telephone or email (for HPs). She then invited them to an interview at a time/place of their choice. Five individuals with ALS, three CGs, and seven HPs participated.

The second round of sampling (theoretical sampling) was guided by emerging analysis and aimed to verify, saturate, and expand the conceptual categories generated from the initial sampling dataset [32]. We then involved an additional five HPs; five individuals with ALS and four CGs.

### 2.4. Data Collection and Analysis

We collected and analyzed data concurrently, and the data were collected through semi-structured, open-ended interviews. We defined three interview guides (according to participant types, as shown in Table 1). We deliberately asked HPs to concentrate on specific cases to prevent narratives from being too general and irrelevant to authentic experiences. When possible, we also asked for perspectives on patients enrolled in the study.

As Charmaz [29] suggested, researchers pre-planned a light, structured interview guide with open-ended questions to let the interviewer concentrate on what the participants were saying and provide a detailed description of their experience (intensive interview). Researchers defined broad foci without applying any prior theoretical framework. As we developed the GTM study, our theoretical sampling also led us to collect data utilizing a revised version of the initial interview guide, asking focused questions related to emerging categories.

All of the researchers received training in qualitative interviewing using a constructivist approach. They were also advised to follow the informants’ interests and thoughts by asking probing open-ended questions, which allowed flexibility for both the participants and interviewers. The researchers conducted interviews alternatively, and these were audio-recorded and then transcribed verbatim immediately afterwards.

After having transcribed 15 interviews with five individuals with ALS, three CGs and seven HPs, SA, ST, GA, EB, CA, FS, LDP and SS began the analysis (open coding) by fracturing data into conceptually labeled codes. SA, EB, LDP, GA, and SS performed the focused coding and grouped the codes into provisional categories (n = 15). We then conducted 12 further interviews during the theoretical sampling process, involving 14 participants; one individual with ALS and her two caregivers participated in the same interview (the patient requested that the CGs be present, and the interviewer agreed because he realized that this would make the patient feel more comfortable). After their transcriptions, SA and SS conducted the theoretical coding, and at this stage, we saturated three categories and renamed them, highlighting the core category.

### 2.5. Memoing and Rigor

Memos [32] were written for each interview and shared within the research group. Memo writing allowed the research team to take the codes apart and analyze their meaning within the interview context [32]. SA and SS wrote memos throughout the research process, with particular attention to developing conceptual categories. SA and SS shared memos and reflections with the research team. Researchers also shared thoughts based on in vivo codes to name the abstract categories.

As to rigor, during the study the researchers tried to avoid data forcing into preconceived codes and categories [32]. The research team comprised nine professionals with different backgrounds (three palliative care specialists, two palliative care nurses, one specialist nurse in education, one psychologist and one bioethicist). The team was supervised by LG (Qualitative Research Methodologist), a non-healthcare professional with a social science background, to limit likely disciplinary pre-assumptions in analyzing data. The team was involved in various capacities in delivering palliative care to patients with neurological conditions.

To assess GTM, Charmaz proposes validity, credibility, originality, resonance, and usefulness as criteria [32]. Credibility was ensured by collecting adequate data to substantiate the conceptualization. The categories were also transversally generated across all the cases. Originality was achieved by using the participants’ words as much as possible during coding. As to resonance, the saturation achieved in our analysis gave us a comprehensive picture of how care is initiated. Finally, we valued the usefulness of this study as this can offer consistent implications for improving care from the outset.

## 3. Results

### 3.1. Study Participants

Thirty-six individuals were contacted, and 29 participants were enrolled in the study (five individuals with ALS, one CG and one HP declined the invitation for reasons that were not disclosed).

Ten individuals with ALS (median age 65 years) were interviewed: eight were suffering from classic ALS and two from bulbar ALS. At the time of the study, four individuals with ALS were being treated with non-invasive ventilation (NIV), and one had undergone a percutaneous endoscopic gastrostomy (PEG). None of the participants had a tracheostomy. The median time since diagnosis was 23 months (ranging from four to 34 months at the interview). Table 2 shows patients demographics/characteristics.

We also interviewed seven CGs (whose characteristics are shown in Table 3) and 12 HPs (please see Table 4).

The final number of interviews was 27. These lasted between 11 and 87 minutes, with an average of 40 minutes. Socio-demographic data were also collected at the end of each interview. Eight interviews were conducted at the homes of the individuals with ALS or CGs, and the remaining interviews were completed in rooms in health facilities.

### 3.2. The Core Category: Off-Beat Interfacing

According to our analysis, the problems of timing in communicating, offering services (namely visits, treatments and devices), and making decisions were at the core of the process of initiating care. Care for individuals with ALS begins with interfacing between HPs and individuals with ALS and CGs, whose concerns, needs, intentions and goals differed in terms of time requirement and need. By naming the core category “off-beat interfacing,” we conceptualized how the demands of HPs did not correspond to the ability of individuals with ALS and CGs to process information, deal with requests, and be at ease in making decisions.

**Figure 1:**
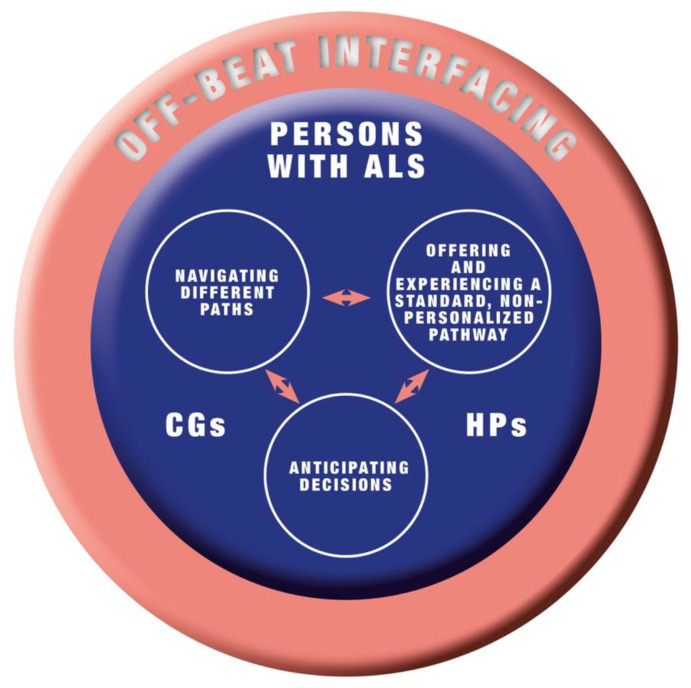
The conceptual model of “off-beat interfacing”.

The core category that connects three categories (see Table 4) was generated from the data analysis: (i) navigating different paths, (ii) offering and experiencing a standard, non-personalized pathway, and (iii) anticipating decisions. The categories (and related sub-categories) have been discussed using participants’ meaningful quotations below. Figure 1 visually summarizes the conceptual model.

### 3.3. Category 1: Navigating Different Paths

Divergent needs emerged in relation to communication of the diagnosis. While individuals with ALS and their CGs wished to receive the diagnosis gradually and with empathy, giving them the time they needed to process the information, HPs felt compelled to communicate the diagnosis and the future course of the disease progression all at once. HPs reported that they wanted to start proposing aids to address future impairments during the communication of the diagnosis. On the other hand, individuals with ALS did not yet feel disabled at this point. HPs acted in the patient’s best interest to determine and plan for the future. Still, individuals with ALS and CGs were unable to comprehend this path, continuing to believe that they would maintain their autonomy for a long time.

Because they needed more time and empathy at that moment, individuals with ALS and CGs perceived the communication of the diagnosis as *“very crude”* (Patient 06). They complained of a lack of humanity and understanding by HPs of the extent to which the patient’s life was being changed. In addition, they asked for a gradual approach to communicating the bad news.

*“A more human attitude, more understanding and with a more… complete vision, right? Of what could be the course of the disease. Therefore, without already giving a glimpse of the final act!”*

(Patient 13)

*“The diagnosis was the initial hit... for me, and it was a good hit! Life changed and not a little... I was expecting something but not so huge, so significant.”*

(CG 11)

From the HPs’ perspective, we found different concerns that did not overlap with those of individuals with ALS and CGs. In terms of communicating the diagnosis, HPs reported their sense of urgency in explaining the disease and its course in full. HPs wanted to ensure there was no doubt, so that the most appropriate treatment choices could be made as soon as possible.

*“I always clarify that this is a disease that cannot be cured, which is called amyotrophic lateral sclerosis because there shouldn’t be too many doubts. The diagnosis should use the name.”*

(HP 24)

For individuals with ALS and CGs, receiving a diagnosis of ALS was a shock, exacerbated by the HPs quickly describing the condition and foreshadowing a dark future. Our data show that individuals with ALS and CGs were fighting to hold onto the idea that their autonomy would not be affected by ALS in the short term, so becoming “a disabled person” was not an option.

*“I realized that I would have been better, but the impact of going around in the chair... you’re telling everyone ‘I’m sick,’ understand?”*

(Patient 09)

Individuals with ALS and CGs wanted to preserve a sense of normality. They talked about the efforts they wanted to make so that everything was as normal as possible without giving in to the difficulties presented by the disease.

*“Your first instinct is to refuse it, to continue doing what you’ve always done. For now I continue with my life, I keep working... they don’t stop me!”*

(Patient 15)

*“We started to find ways to help him, to make him as self-sufficient as possible, autonomous... we tried to keep him autonomous even for eating, even if he was starting to struggle”*

(CG 04)

However, for HPs, initial meetings with individuals with ALS were intended to provide an explanation of what would happen in the future, including in terms of functional impairment. HPs immediately proposed various solutions to overcome disabilities, overlooking the need for individuals with ALS and their CGs to have a sense of normality and their refusal of auxiliary aids.

One HP said he realized he had to “*make them feel disabled ahead of time”* (HP 27).

*“There are families who insist on wanting to normalize everything even when (nervous laughter) the patient is already at an advanced stage”*

(HP 23)

*“The acceptance of the wheelchair, for example, is another very critical moment... they prefer to take ten steps with brutish effort rather than use the wheelchair…”*

(HP 18)

### 3.4. Category 2: Offering and Experiencing a Standard, Non-Personalized Pathway

At the beginning of the care process, being assisted/providing assistance in relation to individuals with ALS means starting tests and consultations, and referring patients to various professionals. For HPs, providing care within a precise pathway was considered to be comforting (as they knew what they had to do) and a means of offering the best care for all individuals with ALS. This conflicted with the perceptions of individuals with ALS and their CGs, who felt it was inappropriate to be subjected to tests and multiple visits.

HPs described how they were at ease in offering a “set”, pre-arranged (HP 26), standardized system of services to be provided to all individuals with ALS. This perception was also strengthened by consideration of the clinical guidelines, which indicate a multidisciplinary pathway as the best possible care.

*“In any case, the organization orders you... the guidelines dictate it! You have to do some tests”*

(HP 19)

Scheduling this large number of visits and medical check-ups was a way for HPs to be very accurate and to provide everyone with the same pathway, even though HPs themselves were aware of the demanding nature of the requests they were making.

*“The activation of the services’ network can sometimes be a bit excessive compared to the patient’s needs, but it’s also true that in a system where you work in a team at different times, to ensure that everything is covered… all patients receive the same. We must provide…”*

(HP 23)

*“We realized that patients get to a saturation point, and they don’t like it.”*

(HP 18)

Some HPs also realized that the pathway, as it was organized, was not meeting the needs of individuals with ALS and CGs.

*“I always say at our meetings that you must give patients time. Sometimes, offering so many things all together to a person… gives me the idea that we almost want to impose some things… not everyone likes the same type of pathway. We’re the ones who must adapt to their needs.”*

(HP 27)

Individuals with ALS and CGs experienced the beginning of care and use of services as a burden. Consultations and tests were overwhelming. They saw a compulsory pathway without fully understanding the rules. Individuals with ALS and CGs asked for more time to use additional services.

*“This pathway, I’ve been… I don’t mean ‘attacked’—that’s not the right word—but so many different people have contacted me… It felt too much… I needed to process it… to be quiet. I felt suffocated... I needed more time.”*

(Patient 15)

*“She’s constantly having visits; you do get a little impatient.”*

(CG 05)

*“Every day, a test or a puncture... a needle”.*

(Patient 10)

### 3.5. Category 3: Anticipating Decisions

The last category we conceptualized refers to decision-making about advance care planning (ACP). Our participating HPs showed great determination to discuss ACP with individuals with ALS. Early on, professionals (respiratory specialists and neurologists) would engage in conversations to get individuals with ALS to decide on future invasive treatments. Professionals initiated these discussions early in the physician-patient relationship, even without actual clinical need, often right after the diagnosis. Talking about invasive/non-invasive treatments clarified what HPs would need to do.

*“I often perceive a sort of anxiety in wanting the advance treatment directives, especially some colleagues directly involved in some procedures. Something that aims more to compensate for the anxiety of being in an urgent clinical condition and not knowing what to do”*

(HP 29)

In doing so, according to individuals with ALS and CGs, HPs did not consider whether this was the appropriate moment. The way HPs initiated this discussion was perceived negatively, resulting in outright rejections of such conversations on ACP.

*“He [the doctor] had a very negative debut because, at the first meeting, he told me: ‘now we have to think about the feeding tube.’ I told him to go to hell! We’ll think about the feeding tube”*

(Patient 13)

*“Clearly, a person who is not totally aware [of the ALS progression] imagines that they will insert a cannula here or there next week! It’s ok preparing her by telling her, ‘Look! It may be that...’. It may be! It seems it was immediate, to do it now... imminent”*

(CG 08)

However, some HPs recognized this “anticipating decisions” as a problem for individuals with ALS.

*“ACP… once I used to start right away and many patients complained because… ‘I still walk. Do I want a tracheostomy? But how do I know?’”*

(HP 18)

*“The patient experienced it as a form of aggression and said: ‘That’s enough! Every time you crucify me because I have to give advance treatment directives’”*

(HP 26)

## 4. Discussion

This core category, “off-beat interfacing,” explains how care for individuals with ALS begins, as negotiated by multiple actors. Three categories inform the process: (i) navigating different paths, (ii) offering and experiencing a standard, non-personalized pathway, and (iii) anticipating decisions. During the first phase of care, HPs, on the one hand, and individuals with ALS and their CGs, on the other, relate to each other in a way that is “out of sync”.

International guidelines [14] must be applied in defining a clinical-care pathway [7]. Such care models will probably satisfy about two-thirds (or slightly more) of patients [7]. The context studied was aligned with the international recommendation. However, how individuals with ALS and their CGs experienced it was critical. The literature notes that individuals with ALS often experience a lengthy diagnostic journey [34] and need personal time to understand and accept it [35]. The communication of an ALS diagnosis is understandably a shock [27]. According to the information reported by individuals with ALS during the interviews (which occurred from 4 to 34 months after the diagnosis), this emotion remains for some time. The way people experience receiving such a diagnosis is personal, and the way in which the diagnosis is delivered plays a central role. Greater satisfaction with the way the diagnosis is delivered to patients relates to the ability/skills of the neurologist and the time spent with that professional when the news is communicated [36]. According to an Australian survey, skills that should be learned and trained relate to responding empathically to the feelings of patients and CGs, sharing the information while suggesting true-to-life objectives, exploring what patients and CGs are presuming or hoping for, and making a plan and following it through [36]. In our study, it emerged repeatedly that the attitudes and approaches of the HPs were very important for individuals with ALS and their CGs.

In this regard, there is a need to improve communication between HPs and individuals with ALS/CGs by recognizing that the time needed for acceptance of the diagnosis by individuals with ALS/CGs can differ, as can the coping strategies they adopt [27,37]. At the same time, diagnosis communication is difficult for HPs [38], whose communication skills come into play and who need improvement [39,40] and effective training programs [41,42,43].

In our study, individuals with ALS described how they wanted to avoid “feeling disabled too early in life”; maintaining prior roles within the family and a sense of normality is one of the preferences of patients that appears most often in the medical literature [16,44,45,46]. Still, because HPs tend to offer standardized care to all individuals with ALS according to the timing of disease progression, and thus functioned as a “machine” that gives “everything to everybody,” they sometimes lacked empathy and respect. Furthermore, this attitude was often perceived as harsh by individuals with ALS/CGs, impairing patient satisfaction, which appears to be greater when they receive tailored services [7].

According to our results, HPs felt obligated to provide a clear explanation of the course and future scenarios of the disease as dictated by symptoms and instrumental examinations. They felt compelled to identify the invasive treatments individuals with ALS would accept or refuse. While this attitude allowed HPs to understand and respect the patient’s wishes, it can fail to consider that there is a “right time” to start these conversations. Literature in this context has demonstrated that ACP tools improve the correspondence between patients’ wishes and HPs’ decisions, with HPs feeling more confident that their decisions are more likely to represent their patients’ preferences [47,48]. However, according to Murray and colleagues [49], appropriate timing for ACP initiation was considered strictly dependent on patient characteristics. The guidelines of the European Federation of Neurological Societies (EFNS) have underlined this point, stressing that ACP should be discussed early with the patient and CGs while respecting the patient’s social and cultural background [14]. According to our findings, “early” or “soon” did not mean “immediately”, but rather “appropriate” for the patients/CGs system, and this highlights what has been conceptualized elsewhere [37] as the “importance of sensitive and timely conveyance of information”. In other words, there is a need to assess the readiness of individuals with ALS and their CGs to have this conversation [50]. However, this point is critical, as it must be considered in balancing against the benefits (i.e., QoL and survival) coming from prompt decisions and interventions. Difficult discussions must be planned to allow individuals with ALS to maximally benefit from the resulting choices. The appropriateness of the timing is at the interplay of medically optimal interventions, informed decision-making, and individuals with ALS/CGs’ readiness and preferences. This study adds an invitation to consider that ACP also involves all the actors’ emotional aspects, which may prevent thoughtful communication (from the HPs’ side) and unbiased comprehension (from the individuals with ALS/CGs’ part). In discussing palliative care for patients with ALS, Mitsumoto and Rabkin [51] recommend that, although care and end-of-life issues are best raised soon after the diagnosis, many of the conversations and decisions can occur when capabilities and functioning start declining.

It should be noted that recent Italian legislation, namely the law on informed consent and advance directives [52] effective from January 2018, prompted HPs to engage in ACP discussions with individuals with ALS. Our participating HPs took this very seriously, often addressing this topic in the first visits immediately after diagnosis.

ACP discussions with HPs about care and treatment options are crucial [53] and individuals with ALS and CGs claim that attention, tact, and sensitivity are important factors [54] as has been noted elsewhere [55]. In this context, AI-computer-based decision aids are promising [56,57,58,59], especially those that combine personalized communication by HPs with intra-familial discussions [60]. Generally, the literature demonstrates how a correct communication process creating a strong therapeutic alliance is fundamental for the subsequent discussion of treatment options [39]. On this issue, indications initially suggested and trialed in oncological care [61,62,63] have also been implemented for neurological palliative care [47,64].

It is clear that our findings and the resulting practical implications call for patient-family-centered care [65]. Patients frequently share decision-making with their CGs [66] throughout the course of the disease [67,68]. Conversely, health services should help individuals with ALS regain control of their care [45] by promoting a patient-family-centered care approach based on the patient’s values and care goals and needs. This caring approach would better support the therapeutic alliance necessary for decision-making [69,70].

Moreover, some HPs in our study stated that the presence of the palliative care team is crucial. A palliative care approach would benefit the entire care process, as it helps patients overcome the feeling of loss they experience with the disease [71,72].

### Limitations and Strengths

This study considered the beginning of the care process for individuals with ALS and their CGs through the interplay of different perspectives. Some methodological limitations should be noted. In terms of the value of the information provided by individuals with ALS and their CGs, a recall bias in some of the participants may be present, given the mean [period?] from diagnosis. Nonetheless, it was essential for us to leave space and time for those actors to process the information about the diagnosis, and to form a relationship with the services and professionals so they could have a richer experience to narrate. We have not returned the interviews to the participants for an accuracy check, or discussed our interpretation with them. However, the findings resulted from teamwork and an internal/external audit.

This GT is contextual, and its results apply to our investigated settings. However, the study provides valuable insights into similar contexts (a multi-professional approach within a public health system). Moreover, it underlines the importance of listening to multiple actors when care dynamics need to be understood. This working hypothesis is transferable to many other care settings and processes.

As a follow-up, we intend to disclose our data and findings to question the clinical care pathway as it is now, and rethink it for individuals with ALS and their CGs.

## 5. Conclusions

The present study describes the psychosocial process concerning how care services for individuals with ALS and their CGs are commenced. HPs and individuals with ALS/CGs expressed different modalities for managing time, meeting needs, and information processing. We encourage HPs to pay more attention to the needs of individuals with ALS and their CGs. While multidisciplinary teamwork can support this, individual communication skills should be improved, exam and visit times should be tailored to clinical conditions, and discussions on end-of-life should consider the time needed to process all of the associated information and feelings. Further research would clarify how patient-family-centered care pathways can be successfully organized.

## Figures and Tables

**Table 1:** The interview guide with examples of questions.

Foci	Individuals with ALS	CGs	HPs
	Typical questions		
Experiencing health services	Could you tell me what services you have met since your diagnosis? Could you tell me how you felt during the interaction with these services?	Which professionals have you met to date? What happened when you went to the service/met any professionals from the service?	Could you tell me what your role is within the health service? Could you please describe a typical pathway for me? Could you tell me how you feel? Do you have a specific case in mind?
Being assisted/assisting within services	Could you tell me what have been the most significant moments so far? What do you need to do now concerning your condition?	Could you tell me how you dealt/are dealing with treatments/decisions? Who do you feel is helping you?	If we concentrate on a specific case, what were the most significant moments concerning patient NN in the diagnostic or therapeutic process? How did you experience that moment?
Relationships within services	Could you please tell me about your expectations from HPs? Which persons do you feel are closest to you in this care pathway?	Could you please tell me about your expectations from HPs? Which people do you feel are closest to you in this care pathway?	With which professionals/services do you share or have shared aspects related to NN?
Closing questions	Do you have any final comments or suggestions for improving the care provided?	Do you have any final comments or suggestions for improving the care provided?	Do you have any final comments or suggestions for improving the care provided?

**Table 2:** Characteristics of individuals with ALS (n = 10).

Code	Gender	Age Range (Years)	Onset Type	Treatment	Months Since the Diagnosis at the Interview
02	F	70–79	classic	NIV	30
03	M	50–59	bulbar	NIV	34
06	F	70–79	bulbar	NIV	17
09	F	50–59	classic	-	33
10	M	60–69	classic	-	6
13	M	60–69	classic	-	12
14	M	60–69	classic	-	4
15	M	50–59	classic	-	11
16	F	70–79	classic	-	30
17	M	60–69	classic	NIV/PEG	33

**Table 3:** Characteristics of CGs (n = 7).

Code	Gender	Age Range (Years)	CG of the Patient (Code)	Relationship with the Patient
01	M	70–79	02	Husband
04	F	50–59	03	Wife
05	F	40–49	-	Nephew
07	M	≥80	06	Husband
08	M	60–69	06	Son
11	F	50–59	10	Wife
12	M	50–59	09	Husband

**Table 4:** Characteristics of HPs (n = 12).

Code	Gender	Age Range (Years)	Role
18	F	60–69	Physical therapist
19	F	40–49	Speech therapist
20	F	40–49	Dietitian
21	F	60–69	Rehabilitation medicine specialist
22	M	30–39	Respiratory specialist
23	F	60–69	Phoniatrist
24	F	30–39	Neurologist
25	F	40–49	Psychologist

## Data Availability

The study documentation is collected and managed by the study coordinator (Azienda USL-IRCCS di Reggio Emilia), and datasets are available on reasonable request.
